# Editorial Note

**Published:** 1915-03

**Authors:** 


### Editorial Note.

**Antityphoid Inoculations.** THE value of antityphoid inoculations has recently been the subject of public discussion and of irresponsible criticism; it is therefore desirable to review the results hitherto obtained, so as to have substantial "foundations for our belief" in the method. We are indebted to Professor Walker Hall for the following notes.

The example set by the British Army Authorities with regard to the prophylaxis of typhoid fever has been copied by a number of nations, and bids fair to prove one of the outstanding advances in modern methods of hygiene. It is possible that the evidence accumulating from the present campaign will result in our following the lead of America and Germany, and instituting compulsory inoculations for the services, etc. The following figures place the validity of the practice on a firm basis. In those instances where the numbers inoculated are available, they are placed under the percentages. Although the data are not strictly comparable, Major Greenwood, jun., of the Medical Statistical Department, states that taking them from the worst possible standpoint, they show "the ratio of cases amongst the uninoculated to be more than six times that amongst the inoculated."

| | Case Incidence. | | Case Mortality. | |
|---|---|---|---|---|
| | Inoculated. | Not Inoculated. | Inoculated. | Not Inoculated. |
| | per cent. | per cent. | per cent. | per cent. |
| United States Army, 1898 | — | — | — | 8.5 / 1,729 |
| ,, ,, ,, 1911 | — | — | 0.001 / 12,801 | — |
| French Army— | | | | |
| Before Inoculation .... | — | 13.82 | — | 2.23 |
| Voluntary Inoculation.. | 3.96 | — | 0.53 | — |
| Compulsory Inoculation | 0.68 | — | 0.11 | — |
| German Army in Africa.. | 5.09 | 9.84 | — | — |
| French Navy .. .. | 0 / 3,107 | 0.8 / 67,843 | — | — |
| British Army in India | 0.98 / 4,502 | 2.54 / 25,851 | — | — |
| British Army—Foreign Stations .. .. .. | 0.53 / 10,378 | 3.04 / 8,936 | 8.9 | 16.9 |
| Various Hospital and General Statistics .. | 2.25 | 5.75 | 12 | 21 |

The seat of injection seems to vary with the individual, but the general consensus of opinion points to the scapular and deltoid areas as yielding the least discomfort to the patient.

The dosage also has not been uniform. For ladies and young people there are many who prefer three or four doses of 100, 250, 500 and 1,000 millions, instead of the customary two injections. After the injection it has been found necessary to diminish muscular work for twenty-four to forty-eight hours, if ill-effects are to be uniformly avoided. The local and after-effects depend to some extent upon the methods used in preparing the vaccine. The ideal vaccine is still the pursuit of many research workers.

The appearance of antibodies in response to the injections does not seem to be generally realised, and cases have occurred in which a subsequent Widal reaction has led to the diagnosis of enteric fever. In one of their experiments, Pfeiffer and Kolle found that on the eleventh day after injection of dead typhoid bacilli the agglutinating powers of the patient's blood rose five to fifty times above that of normal blood, and the bacteriolytic power had been increased five-fold. Perhaps it may some day become necessary to determine the titre of the blood as a test of immunity. During an attack of typhoid fever the agglutinating value of the blood is inconstant and tends to fall ; after preventive inoculations it rises and remains steady. Hence a number of determinations of the agglutination titre may yield useful information in doubtful cases. If the patient has been inoculated against enteric fever the agglutination tests should include other organisms than the B. typhosus, and blood cultures should be made as a routine procedure.

Much has been written as to the period of the resistance obtained. Many place it as long as two years. Others say six months. In the present state of knowledge, it appears that the patients should be inoculated whenever they return to England.

There are still those who lend their influence to dissuade the soldier or civilian from safeguarding himself by prophylactic injections. It must be difficult for those who are in the midst of this kind of work to appreciate fully the attitude of such dissentients. The laboratory worker is accustomed to test his opinions and findings by personal experiments, and to record actual results. If those who oppose this form of inoculation would personally put their tenets to the test by exposing themselves to contagion in trench, field or hospital, no one more than the scientist would be ready to accord credit for their work, and to consider their figures without prejudice. Such a course might lead to an advance in knowledge. The mere crying out on placards and pamphlets leads nowhere.

Owing to the possibility of paratyphoid infections, either as single infections or as an addition to that of the typhoid bacillus, it has been suggested that mixed prophylactic vaccines should be used. This proposal merits serious attention, especially as a paratyphoid attack may be ascribed to a mild form of typhoid, unless the agglutination tests are applied more fully than is now customary.

---

### Therapeutic Notes.

